# Different Host Plants Distinctly Influence the Feeding Ability of the Brown Citrus Aphid *Toxoptera citricida*

**DOI:** 10.3390/insects12100864

**Published:** 2021-09-24

**Authors:** Runa Zhao, Chengxu Wu, Yingqin He, Chun Yu, Jianfeng Liu, Taisheng Li, Changyong Zhou, Wenlong Chen

**Affiliations:** 1 Guizhou Provincial Key Laboratory for Agricultural Pest Management of the Mountainous Region, Institute of Entomology, Scientific Observing and Experimental Station of Crop Pest in Guiyang, Ministry of Agriculture, Guizhou University, Guiyang 550025, China; zhao21373@163.com (R.Z.); yu16719@126.com (C.Y.); jfliu3@gzu.edu.cn (J.L.); 2 College of Forestry, Guizhou University, Guiyang 550025, China; cxwu3@gzu.edu.cn; 3 College of Tea Science, Guizhou University, Guiyang 550025, China; yqhe1@gzu.edu.cn; 4 Citrus Research Institute, Southwest University/Chinese Academy of Agricultural Sciences, Chongqing 400712, China; litaisheng@cric.cn (T.L.); zhoucy@cric.cn (C.Z.)

**Keywords:** *Toxoptera citricida*, electropenetrography, host plants, feeding behavior, plant resistance

## Abstract

**Simple Summary:**

The brown citrus aphid, *Toxoptera citricida,* is an important pest of citrus and causes serious damage in the main production areas. Host plant resistance is an environmentally friendly method to manage aphid infestations and is becoming increasingly important as aphids develop greater resistance to insecticides. The aim of this study was to assess *T. citricida* resistance on seven widespread and common hosts using electropenetrography combined with a population development test. We showed that the feeding parameters of the brown citrus aphid differed significantly depending on the host plants. *Toxoptera citricida* spent more time in the pathway stage and less time in the phloem stage on Eureka, while the opposite was observed on Yuzu and Rough Lemon. Measurements of population development of aphids showed that on the Eureka, aphids developed more slowly. Our data suggest that different host plants distinctly influence the ability of *T. citricida* to feed. The brown citrus aphid did not prefer to feed on Eureka Lemon compared to the other six host plants.

**Abstract:**

Piercing–sucking insects are important crop pests, and an understanding of their feeding behavior and population development plays a crucial role in studying insect population dynamics and crop resistance. In our study, we examined the probing behavior of the brown citrus aphid, *Toxoptera citricida*, using electropenetrography and assessed its population development after 8 days on seven host plants: Yuzu, *Citrus junos* Sieb. ex Tanaka; Rough Lemon, *C. jambhiri* Lush.; ‘Luofu’ kumquat, *Fortunella margarita* Swingle; ‘Olinda’ valencia orange, *C. sinensis* (L.) Osbeck; ‘Yanxiwanlu’ Ponkan, *C. reticulata* Blanco; ‘Rohde Red’ valencia orange, *C. sinensis*; and ‘Eureka’ lemon, *C. limon* (L.) Osbeck. The results demonstrated that probing by the brown citrus aphid differed significantly according to the target hosts. *Toxoptera citricida* produced significantly more pathway activities on Eureka than on Rough Lemon and Yuzu. *Toxoptera citricida* spent more time from the first probe to first salivation into phloem sieve elements on Eureka compared to Yuzu. In addition, the total duration of ingestion from sieve cells of each aphid in the phloem-feeding phase was shortest on Eureka, and this was significantly shorter than that on Yuzu, Rough Lemon, Luofu, and Olinda. The population number of *T. citricida* on Eureka after 8 days was significantly lower than that on the other hosts. Overall, Eureka was found to have obvious resistance to *T. citricida*, whereas Yuzu and Rough Lemon were susceptible host plants. These results provide a theoretical basis for exploring aphid-resistant fruit tree resources using resistant varieties.

## 1. Introduction

Citrus is a globally important economic fruit tree. It is the most widely grown fruit tree in the subtropical regions of China and is often attacked by various insect pests [1,2]. Citrus aphid species, such as *Toxoptera citricida* (Kirkaldy) (Hemiptera: Aphididae), *Aphis gossypii* Glover (Hemiptera: Aphididae), and *A. citricola* Van der Goot (Hemiptera: Aphididae) are important and serious pests, and their infestation reduces citrus production because they consume nutrient compounds, for example, from citrus flush shoots and young leaves, in addition to acting as vectors of various pathogenic plant viruses [3,4]. Among them, the brown citrus aphid *T. citricida* is one of the most serious pests of citrus in the world because it is currently the most efficient vector of the *Citrus tristeza* virus (CTV) [5,6]. CTV is a phloem-limited closterovirus transmitted in a semipersistent manner and via vegetative propagation of the infected budwood. It is considered a devastating and destructive threat to the citrus industry worldwide [7,8,9]. The presence of *T. citricida* expedites the spread of CTV in the field from one citrus to another citrus [10], either located in the same citrus orchard or more distantly, thereby affecting the safe production of citrus varieties. 

At present, the application of pesticide is the main approach for controlling aphids [11,12,13]. However, with the expansion of the citrus industry, the widespread use of chemical controls has resulted in increasing incidences of insecticide resistance. Considering the various negative effects of the application of conventional pesticides, an alternative strategy for aphid control is the development and application of integrated pest management (IPM) and the utilization of host plant resistance from the citrus germplasm bank. Reports on the screening of aphid-resistant hosts and studies clarifying the mechanisms of such resistance among different hosts have increase; for instance, a comparative analysis of resistance to the aphid *Eriosoma lanigerum* (Hausmann) (Hemiptera: Aphididae) in the main apple cultivars in China [14], and resistance and tolerance of ten carrot cultivars to *Dysaphis crataegi* Kalt (Hemiptera: Aphididae) [15]. However, published reports on citrus host resistant to *T. citricida* are scarce and contain only fragmentary data on aphid population development [16,17,18].

Studying the probing and feeding behavior of aphids is important to understand crop resistance. The Electrical Penetration Graph (EPG) technique is a useful tool that provides detailed information on the probing and feeding behaviour of piercing-sucking hemipteran insects in real-time. A series of characteristic aphid EPG waveforms are associated with stylet tip positions inside the plant tissue and with specific probing and feeding activities [19,20,21,22,23,24]. This excellent technique has been widely applied to determine the relationship between feeding activities and plant resistance to aphids such as *Nasonovia ribisnigri* (Mosley) (Hemiptera: Aphididae) [25], *Rhopalosiphum maidis* (Fitch) (Homoptera: Aphididae) [26], *A. gossypii* [27,28], *T. aurantii* (Boyer de Fonscolombe) (Hemiptera: Aphididae) [29], *Myzus persicae* (Sulzer) (Homoptera: Aphididae) [30,31], *Macrosiphum euphorbiae* (Thomas)(Homoptera: Aphididae) [30], *Schizaphis graminum* (Rondani)(Homoptera: Aphididae) [32], *Sitobion avenae* Fabricius (Hemiptera: Aphididae) [33], and *E. lanigerum* [14].

In the current study, we recorded the stylet penetration behavior of *T. citricida* on seven host plants to assess the resistance level and the relative resistance of different hosts to the aphid. This experiment provides a theoretical basis for exploring aphid-resistant fruit tree resources using resistant species.

## 2. Materials and Methods

### 2.1. Test Plants and Maintenance of Aphid Colonies 

Seven host plants of citrus commonly found in the southwest of China were studied: Yuzu (*Citrus junos* Sieb. ex Tanaka), Rough Lemon (*C. jambhiri* Lush.), ‘Luofu’ kumquat (*Fortunella margarita* Swingle), ‘Olinda’ Valencia orange (*C. sinensis* (L.) Osbeck), ‘Yanxiwanlu’ Ponkan (*C. reticulata* Blanco), ‘Rohde Red’ Valencia Orange (*C. sinensis*), and ‘Eureka’ lemon (*C. limon* (L.) Osbeck). 

The host plant seeds were provided by the Citrus Research Institute, Southwest University on 8 January 2020. After about 3 months, citrus seedlings of a similar size were collected, and the seedlings were transplanted into plastic pots (D 16 cm × H 28 cm). They were then held in an artificial climate chamber (25 ± 3 °C, 65 ± 5% RH, L 14:D 10 photoperiod) until they reached 20–40 cm in height, and fresh leaves of whole plants were used for the experiments. 

*Toxoptera citricida* aphids were collected on *C. unshiu* Marc. leaves from the teaching experimental farm of Guizhou University in September 2017. These aphids were reared on 2-year-old citrus seedlings for several generations inside an insect-proof cage (50 cm × 50 cm × 50 cm) kept in climate-controlled chambers at 25 ± 1 °C, 75 ± 2% RH, and an L 14:D 10 photoperiod. An apterous adult female from the stock colonies was transferred to each of the seven host plants for each experiment. After three generations of such rearing, adult aphids within 4 h of molting were used for EPG studies. Aphids were only used once.

### 2.2. EPG Recording and Analysis

The feeding behavior of aphids was recorded using an EPG-8 dd (Giga-8 dd; EPG Systems, Wageningen, The Netherlands) in a Faraday cage. Before recording, the aphids were transferred into a clean Petri dish using a moistened camel hair paintbrush. The terminal of a gold wire (2–3 cm long × 12.5 μm in diameter) was glued to the pronotum of an aphid with a water-soluble conductive silver glue. Wired aphids were placed in a clean Petri dish for 1 h under fasting conditions while adapting to the stress of the wire tether. Seven host plants were randomly arranged in Faraday cages, and leaves were turned over and fixed by a membrane of stretched Parafilm (Pechiney Plastic Packaging, Menasha, WI, USA). A plant electrode was inserted in the moistened soil around the citrus seedling, and the insect electrode was connected with the insect biological current amplifier. When the aphids were placed individually on the abaxial surface of the leaves and pierced the plant’s tissue, the circuit was completed. The feeding behavior of the aphids was transformed into different waveforms and displayed on the screen. Stylet software including Stylet+d and Stylet+a, is available at www.epgsystems.eu (accessed on 16 September 2021). Stylet+d software was used to record waveforms, and Stylet+a software was used to identify and manually annotate the waveform events according to Tjallingii [34], Spiller et al. [35], Tjallingii and Hogen Esch [20], and Garzo et al. [36]. Waveform np is associated with nonprobing behavior, and its shape is a flat line. Waveform C includes waveforms A, B, and C, and represents the stylet intercellular space, where insects exhibit mechanical stylet penetration and gelling saliva secretion. Waveform pd is the potential drop waveform, which represents an intracellular stylet puncture. Waveform pd includes two different pds, the standard pd and the phloem-pd, the former of which is associated with stylet punctures in the pathway phase and the latter of which is associated with stylet punctures in sieve elements and companion cells. Waveform E1 represents watery salivation into phloem sieve elements at the beginning of the phloem phase, and waveform E2 is correlated with passive phloem sap uptake from the sieve elements. Furthermore, waveform G represents the active intake of xylem sap, and waveform F represents derailed stylet movements. The analyzed waveforms were summarized using an EPG analysis worksheet 4.4.3 software [37]. All experiments were performed in a climate-controlled chamber (25 ± 2°C).

For each host plant, the stylet penetration activities were recorded for 35 aphids per treatment and for 6 h per aphid. Aphids that did not complete the 6 h recording period were not included in the data. The sample size per host was Yuzu = 25, Rough Lemo*n* = 21, Luofu = 15, Olinda = 24, Yanxiwanlu = 25, Rohde Red = 25, and Eureka = 15.

### 2.3. Population Development

For all host plants, resistance to *T. citricida* was tested in a no-choice experiment. One pot of each host plants tested was placed in a plastic tray (D = 18 cm) throughout the experiment. It was filled with water (2 cm deep) to prevent aphid movement from plant to plant. Five recently molted (1–2 days old) adult apterae of *T. citricida* were transferred to the first fully expanded leaf of one plant of each host plant (six plants per host). The assays were conducted in a climate chamber at 25 ± 3 °C, 65 ± 5% RH, L 14:D 10 photoperiod. The total numbers of aphids, adults, and nymphs were counted on day 8 of the experiment. 

### 2.4. Data Analysis

In sequential parameters, when time to waveforms related to phloem phase (E1 or E2) was calculated, the time from the first probe until the end of the recording was used if no phloem phase occurred. Differences in the EPG results among host plants were evaluated in IBM SPSS Statistics v.26.0 (SPSS, Chicago, IL, USA). For all ANOVA analyses, residual plots were checked for normality (Shapiro–Wilk test) or homoscedasticity (Levene’s test) of the residuals. In the case of the absence of normality, the data were transformed with a log10(x + 1) transformation. Even after these transformations, residuals did not conform to a normal distribution. Thus, all data were analyzed using the nonparametric Kruskal–Wallis test. If the difference was significant, we used post hoc multiple comparisons of mean ranks for all groups (Duncan’s test). The mean number of aphids on the different hosts were compared using one-way ANOVA with Fisher’s Protected LSD test.

## 3. Results

### 3.1. Overall Characteristics of EPG Recordings

**Figure 1:**
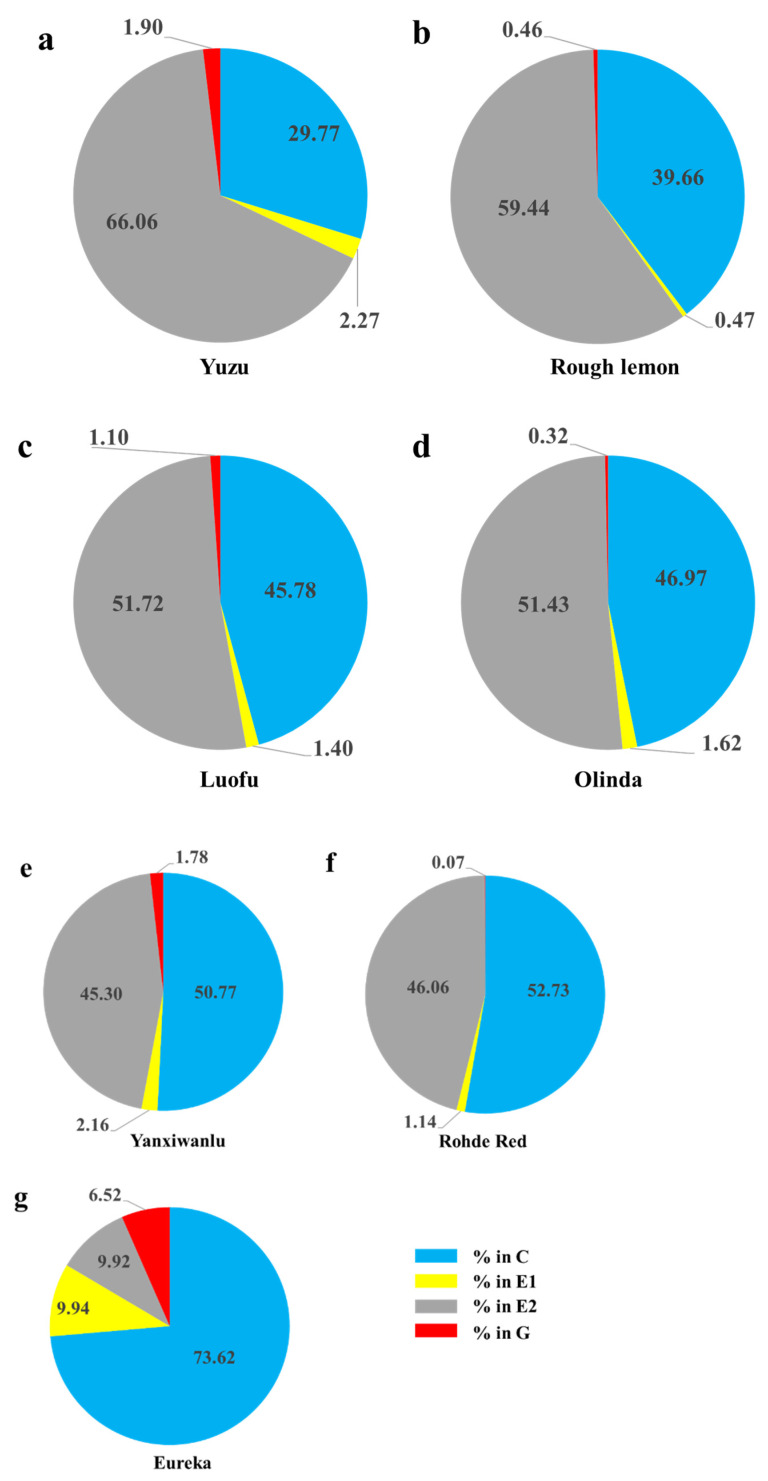
Total percentage of time per individual *Toxoptera citricida* spent ingesting in the pathway, xylem and phloem phases (the sizes of each pie slice) on seven host plants: pathway waveforms (C), phloem salivation (E1), phloem ingestion (E2), and xylem activities (G). (**a**) Yuzu, *n* = 25; (**b**) Rough Lemon, *n* = 21; (**c**) Luofu, *n* = 15; (**d**) Olinda, *n* = 24; (**e**) Yanxiwanlu, *n* = 25; (**f**) Rohde Red, *n* = 25; (**g**) Eureka, *n* = 15.

*Toxoptera citricida* showed six distinctly different EPG waveforms (np, C, pd, E1, E2, and G) on all seven hosts. There was no occurrence of F during the 6-h recording time. *T. citricida* reached the phloem on all seven host plants. Waveforms C and E2 appeared at higher proportions in almost all EPG records, while waveform G appeared less frequently in proportions ranging from 0.07% and 6.52% (Figure 1). Most of the general variables presented significant differences among the seven host plants.

### 3.2. Pathway Activities

The percentage of pathway activities ranged from 29.77 to 73.62% on the seven hosts. The waveform C of *T. citricida* adults on Eureka (73.62%) was about two times higher than that on Rough Lemon (39.66%) and Yuzu (29.77%) (Figure 1). The probing duration time per insect was the longest on Yuzu and shortest on Eureka, although there was no significant difference between them (Table 1). The number of probes per insect on Yuzu was significantly lower than that on Olinda and Rohde Red, and this parameter on Luofu was significantly lower than that on Rohde Red. Probing duration per probe per insect on Yuzu was significantly longer than on Olinda and Rohde Red (Table 1). 

The time to the first probe on Rough Lemon was significantly greater than that on Yuzu, Luofu, Yanxiwanlu, and Rohde Red. Rohde Red had the highest number of short probes, and this was significantly higher than that for Yuzu (Table 2). The brown citrus aphid made significantly more penetrations until the first E1 on Rohde Red and Olinda than that on Yuzu. Additionally, *T. citricida* adults spent more time from first probe to first E1 on Eureka than Yuzu. Moreover, the time from first probe to first E2 (min) was the longest on Eureka, which was significantly longer in comparison with Rough Lemon, Rohde Red, and Yuzu. However, there were significant differences among the seven host plants for variables measured over more than 10 min regarding E2 (Table 2).

The total duration time of waveform C per aphid (WDI) on Rohde Red and Eureka was significantly longer than on Yuzu and Rough Lemon. The total number of waveform C and np per aphid was significantly lower on Yuzu compared to Rohde Red and Olinda. Compared to Rohde Red, the total number of short probes per aphid on Yuzu was significantly lower. The pd waveform duration per event per insect on Luofu was significantly higher than on Rough Lemon, Eureka, and Olinda. The total number of pd waveforms per aphid was significantly higher on Eureka than on Yuzu and Rough Lemon (Table 3). 

### 3.3. Phloem and Xylem Activities

Adults of *T. citricida* often ingested sap from phloem tissue (47–96% of insects recorded performed E2) on the seven hosts. When the aphid was feeding on Yuzu and Rough Lemon, the proportion of E1 + E2 waveforms (feeding on phloem) was higher (68.33 and 59.91%, respectively) than when feeding on other plants, especially Eureka (19.86%). For waveform G (%), no differences were observed among the seven host plants (Figure. 1).

However, the aphids on Eureka spend more time salivating in the phloem (E1) than did aphids on other plants (Table 3). The total number of E1 and E2 per insect did not differ among hosts tested (Table 3).

### 3.4. Population Development

**Figure 2:**
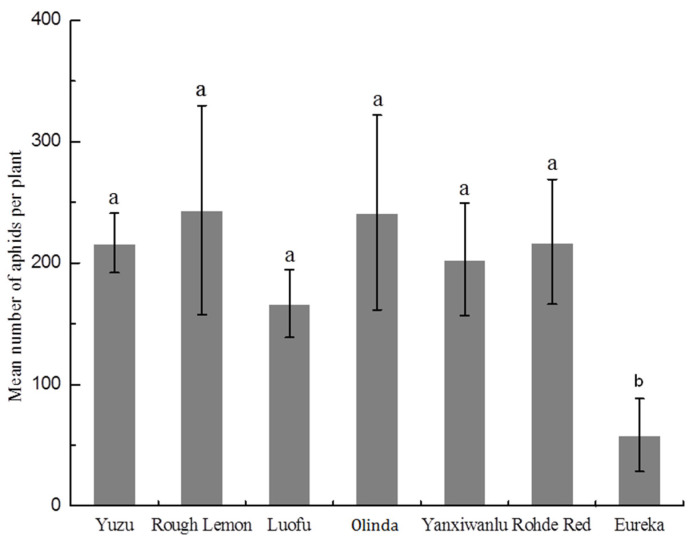
Mean number of aphids per plant on seven host plants after 8 days in a non-preference test under nonchoice conditions. Means ± SE (*n* = 6) marked with the same letter do not differ significantly (*p* > 0.05) based on one-way ANOVA with Fisher’s Protected LSD test.

The mean number of aphids remaining on the plants was lowest on Eureka after 8 days on the hosts (Figure 2). Eureka hosted significantly fewer aphids (*p* < 0.0001) than did the other host plants.

## 4. Discussion

*Toxoptera citricida* is an important pest of citrus, which sucks the phloem sap and transmits *Citrus tristeza virus* [5]. EPG techniques have been very useful in revealing details of the subtle differences of insects probing in different cell layers of plants [38]. Our results obtained for the detailed *T. citricida* behavioral trajectory on living leaves had practical implications because the probing behaviors of the brown citrus aphid were strongly correlated with crop aphid resistance factors and screening of resistant species. It has been reported that experiments on population development provide a more comprehensive assessment of the effect of resistance on aphid performance [30]. In the current study, the behaviors of *T. citricida* in accepting or rejecting a host plant on seven host plants were elucidated by this EPG technique. Here, six waveforms (np, C, pd, E1, E2, and G waveforms) were observed, which was consistent with our previous studies with EPG measurements in *C. unshiu* [39]. Feeding behavior and population development experiments showed Eureka exhibits obvious resistance to *T. citricida*, while Yuzu and Rough Lemon were susceptible host plants. Field evaluation of citrus germplasm for resistance to the black aphid *T. aurantii* showed that there was a significantly lower mean number of aphids per flush for Eureka on 78 citrus varieties/hybrids studied [16]. Further research should focus on whether Eureka is resistant to other citrus aphids. Moreover, the differences detected in specific EPG parameters also demonstrated that epidermal, mesophyll, and sieve element factors on different host plants were important in *T. citricida* stylet penetration behaviors.

Aphids first identify the plant surface and assess plant suitability when settling on the leaves. Leaf surface/epidermis structural traits (trichomes, surface waxes, hardness or thickness of leaf tissues) and chemical cues (emission of volatiles, many forms of terpenes) easily disturb insects feeding behaviors [15,40,41]. Defensive structures in the epidermis may include oil glands and silica inclusions called raphides [42]. In our study, we showed that the plant surface strongly influences aphid feeding behaviors. Time to the first probe is related to resistance on the surface of the leaves. We found that the brown citrus aphid took a longer until its first probe on Rough Lemon, Olinda and Eureka compared to other host plants. Glandular secreting trichomes is known to produce a variety of volatile and nonvolatile plant secondary metabolites including acylsugars, terpenoids, phenylpropanoids and flavonoids [43]. Among the glandular trichomes, types IV and VI are associated with high-level resistance to aphids and whiteflies in Solanum spp. [44,45]. Acylsucrose secreted by the type IV glandular trichomes is an antixenotic factor that deters whitefly settling and delays stylet penetration by whiteflies on tomato plant genotype ABL 14–8 leaves (resistant to *Bemisia tabaci* (Gennadius) (Hemiptera: Sternorrhyncha)) [46]. Furthermore, this structure modifies the innate behavior of *B. tabaci* for settling and feeding on the abaxial surface of tomato leaves [46]. The VOCs from *Ocimum basilicum* L. and *Tagetes patula* L. also reduced sustained feeding and increased nonproductive probing and searching [47]. The surface resistance characteristics of Rough Lemon, Olinda, and Eureka mentioned above remains to be elucidated. However, in the current study, leaf surface structural traits likely did not negatively impact aphid populations, with Rough Lemon and Olinda supporting higher populations than did Eureka. *T. citricida* took a longer time to start the phloem phase on Eureka than that on the other host plants studied, perhaps because of differences in the thickness of the epidermal cuticle. This was shown to affect the probing of one whitefly species on lemon leaves, for which penetration was less successful or failed when the cuticle was thick [48].

In aphids, stylet penetration, specifically phloem sieve element penetration, is essential for host plant acceptance and rejection [49]. Short probes (C < 3 min) are usually confined to the epidermal or mesophyll cells of leaves, which are important indicators of gustatory cues because aphids will ingest small amounts of plant cell contents. Aphids most likely use the cues to navigate through the plant and use, for instance, sugar concentrations to determine whether they are in sieve elements or in other cells of the plant [38]. The pathway phase was longer for *T. citricida* on Rohde Red and Eureka than on the other host plants. At the same time, the total duration time of nonpenetration, and the number of probes per insect, were higher on Eureka. These results indicated that the epidermis/mesophyll has resistance factors and reflects the difficulties in selecting an appropriate feeding site when exposed to Eureka leaves. More extended nonprobing periods and fewer number of short probes and probes have been reported for soybean aphids (*Aphis glycines* Matsumura (Homoptera: Aphididae)) on the stem than on the adaxial and abaxial leaf surfaces. Maybe denser and longer nonglandular trichomes on stems than on either leaf surface interrupts and slows down feeding by herbivores [50]. Factors of mesophyll tissue resistance include cell wall resistance to the physical and chemical penetration of the pest insects [51]. In order to elucidate the cause of this prolonged pathway, further studies will be necessary. 

Following the pathway phase, the activity of the aphids in the phloem may be initiated. Salivation into phloem phase (E1) always precedes ingestion from phloem sap (E2), which plays a vital role in overcoming plant defense mechanisms. In our study, the total percentage and total duration time of E1 per aphid was higher on Eureka than on other hosts tested. E1 waves were observed to alternate with short E2 waves, or otherwise E1 waves were followed by a stylet pathway phase. These types of difficulties in initiating ingestion from the phloem have also been reported for *Cucumis melo* L. genotypes TGR-1551 or AR5, and the green peach aphid *A. gossypii* [27,28]. The E1 waveform may involve ingestion of small quantities of phloem sap to a gustatory organ in the aphid foregut [52]. Chemical solutes of the phloem sap may therefore be detected; the insect may be making multiple gustatory samples during this longer period of sieve element evaluation to gain more reliable information regarding the quality of its feeding site, likely evoking the rejection of sieve elements in resistant plants. It is possible that there are resistant secondary substances in the phloem of this variety, which is consistent with previous studies in *Acyrthosiphon pisum* (Harris) (Hemiptera: Aphididae), which had a high proportion of salivation during phloem activity on barely acceptable forage legumes [53]. Maybe Eureka produces a stronger defense response when fed on by aphids than do other plants. Once the aphids pierce the sieve tube, the main defense response of the plant is triggered, i.e., sieve tube occlusion. Plants try to prevent sap loss by depositing callose. To overcome this defense mechanism, aphids secrete watery saliva that contains calcium-binding protein [54]. Caillaud and Niemeyer compared sap exudation from excised stylets of the grain aphid, *Si. avenae*, feeding on aphid resistant and aphid susceptible wheat lines [55]. They found reduced exudation from the resistant lines, suggesting an enhanced phloem sealing system in resistant plants that includes callose deposition in sieve elements.

Time spent phloem sap feeding is the most direct parameter reflecting host plant suitability for a piercing–sucking insect [56]. The total duration time of ingestion of phloem sap (E2) on Eureka was the shortest, and significantly lower than that on Yuzu, Rough Lemon, Luofu, and Olinda during our 6 h experiment. Similarly, fewer E2 events were observed on Eureka. This failure of the aphids to feed on the phloem during six recording hours could be related to the significantly longer time spent in nonprobing activities and the pathway phase. However, when accessing the phloem, fewer aphid individuals ingested sap on Eureka for longer than 10 min than on other hosts. Probably the aphids found a type of deterrent or a physical barrier in the phloem cells that stopped ingestion and reduced further phloem feeding. The significant reduction in feeding time in the phloem should logically result in a reduction in nutritional resources and lead to a decrease in the aphid’s fecundity. The mean number of aphids remaining on the plants was lowest on Eureka after 8 days on the hosts compared with other hosts, and significantly so, which was also consistent with our results of feeding behavior by EPG. It was clearly demonstrated that Eureka was highly resistant to *T. citricida*. This resistance can be important for limiting the transmission of phloem-restricted viruses. This is because in the semipersistent or persistent transmissions, virus acquisition from sieve elements can occur within minutes, but the transmission efficiency increases with prolonged insect feeding [57]. *Bemisia tabaci* spent more time in nonprobing activities and showed a reduced ability to start probing on the tomato breeding line ABL 14-8 versus the variety Moneymaker, which resulted in a reduced ability to reach the phloem. Interestingly, the presence of type IV glandular trichomes and the production of acylsucrose on the ABL 14-8 were effective in significantly reducing the primary and secondary spread of tomato yellow leaf curl disease (TYLCD) caused by the *Tomato yellow leaf curl virus* (TYLCV) [58]. Previous studies have shown that the application of targeted chemicals deterred pests from probing and feeding, thus also limiting virus spread. Some jasmonates such as cis-jasmone, dihy-drojasmone, and basically all derivatives of these natural compounds, are responsible for the extended time of the nonprobing of *M. persicae* on *Brassica rapa* subsp. *pekinensis*, which may contribute to the limitation of the transmission of semi-persistent and persistent viruses [21,59]. Hence Eureka, on which a small percentage of aphids reach sustained phloem ingestion, and on which E2 has a shorter duration (phloem factor resistance), will delay and reduce the chances of acquisition and spread of a phloem-restricted virus such as CTV.

On the seven host plants, during the 6-h recording time, *T. citricida* spent 0.07–6.52% of the time on xylem sap ingestion and 19.86–68.33% on phloem sap. Similarly, during its adult and nymphal stages, *T. citricida* fed on the phloem rather than xylem of *C. unshiu* [39]. The brown citrus aphid appeared in the highest percentage of xylem ingestion (G) on Eureka, although there were no differences among species and the percentage of G waveform was much less compared to other waveforms. Similarly, previous work observed that *M. persicae*–barley interactions (poor-host interaction) were characterized by an increase in xylem ingestion, compared with *M. persicae*–Arabidopsis (host) interactions [60]. The insects tended to dedicate a higher percentage of their total probing activities to xylem sap ingestion when on resistant plants compared with susceptible plants [61]. Xylem feeding has been shown to increase in occurrence and duration after periods of starvation in aphids [35]. In our study, most aphids on Eureka did not reach sieve elements to acquire key nutrients, resulting in an increased total xylem duration due to the aphids starving.

In conclusion, it is important that more insect-resistant host plants are cultivated to prevent further outbreaks of citrus aphids. Assessing together the parameters we measured and analyzed, we infer that Eureka is a *T. citricida*-resistant plant. The location of protection against aphids is distributed in different layers of tissues (surface, epidermis, mesophyll tissues, and phloem elements) in different citrus species. In future studies, we will conduct microscopic observations and electropenetrography to determine these factors and possible chemical cues [62]. A combination of laboratory and long-term field experiments should be conducted to identify host plants that consistently show high resistance to citrus aphid infestation. Through their association with the resistance genes of resistant host plants, it should be possible to identify quantitative trait loci related to the resistance against citrus aphids.

## Figures and Tables

**Table 1:** The durations (min) and counts of probing variables recorded using an EPG study of *Toxoptera citricida* on seven host plants.

Variables/ per Insect	Host Plants								
	Yuzu	Rough Lemon	Luofu	Olinda	Yanxiwanlu	Rohde Red	Eureka	H	P
Probing duration	343.40 (306.47–350.62)a	319.32 (257.64–334.59)a	285.24 (265.18–356.57)a	305.42 (228.82–335.80)a	310.31 (277.87–336.36)a	306.37 (273.98–330.82)a	281.08 (241.45–351.41)a	13.04	0.042
Number of probes	5.00 (2.50–10.00)c	9.00 (6.00–13.00)abc	7.50 (2.75–11.25)bc	15.00 (7.00–20.00)ab	9.00 (4.00–13.50)abc	15.00 (11.50–22.00)a	12.00 (5.00–21.00)abc	26.86	0.000
Probing duration per probe	68.81 (28.13–145.45)a	35.48 (18.65–58.16)ab	38.12 (24.63–133.75)ab	16.35 (12.90–43.99)b	33.40 (17.44–81.44)ab	20.96 (13.01–28.72)b	22.48 (12.95–70.28)ab	23.39	0.001

Values presented as medians (interquartile range) as well as H-statistics and Chi-square probability. Different letters in rows show significant differences between treatments at *p* < 0.05 (Kruskal–Wallis test and post hoc multiple comparisons of mean ranks for all groups Dunn’s test).

**Table 2:** Sequential EPG parameters of stylet penetration activities.

Variables	Host Plants								
	Yuzu	Rough Lemon	Luofu	Olinda	Yanxiwanlu	Rohde Red	Eureka	H	P
Time to 1st probe (s)	0.00 (0.00–80.69)b	51.99 (35.11–83.30)a	0.00 (0.00–97.24)b	20.76 (0.00–43.51)ab	0.00 (0.00–25.10)b	0.00 (0.00–4.94)b	16.46 (0.00–59.07)ab	29.36	0.000
Number of short probes (C < 3 min)	4.00 (1.00–5.50)b	6.00 (3.00–8.00)ab	2.50 (0.00–5.75)ab	8.00 (4.00–11.00)ab	4.00 (1.50–7.50)ab	7.00 (3.50–13.50)a	5.00 (2.00–11.00)ab	17.89	0.007
Number of probes to the 1st E1	5.00 (2.00–8.00)b	9.00 (5.00–11.50)ab	5.50 (1.75–11.25)ab	11.00 (7.00–19.00)a	7.00 (4.00–9.00)ab	12.00 (9.00–17.50)a	7.00 (5.00–19.00)ab	26.74	0.000
Time from 1st probe to 1st E1(min)	65.69 (31.05–108.68)b	118.47 (79.65–192.00)ab	126.45 (47.80–242.36)ab	144.90 (83.30–239.03)ab	116.83 (89.44–191.86)ab	108.69 (76.79–221.82)ab	161.47 (124.66–319.61)a	14.57	0.024
Time from 1st probe to 1st E2(min)	66.61 (32.32–111.02)b	119.14 (80.56–207.55)b	166.22 (49.45–295.14)ab	146.04 (84.30–249.45)ab	121.49 (90.80–216.76)ab	109.42 (77.85–222.89)b	357.55 (239.46–359.95)a	27.93	0.000
% E2>10min in E2	100.00 (100.00–100.00)a	100.00 (100.00–100.00)a	100.00 (83.33–100.00)a	100.00 (100.00–100.00)a	100.00 (100.00–100.00)a	100.00 (100.00–100.00)a	12.50 (0.00–50.00)b	26.92	0.000

Values presented as medians (interquartile range). H-statistics and Chi-square probabilities are presented. Different letters in rows indicate significant differences between treatments at *p* < 0.05 (Kruskal–Wallis test followed by post hoc multiple comparisons of mean ranks for all groups—Dunn’s test).

**Table 3:** Nonsequential EPG variable values for *T. citricida* probing behavior on seven host plants during a six-hour recording.

Variables	Host Plants	ppw	NWI	P	WDI	P	WDEI	P
np	Yuzu	25/25	5.00 (2.00–9.50)c	0.00	16.60 (9.38–53.53)a	0.04	2.99 (2.25–53.53)a	0.36
	Rough Lemon	21/21	9.00 (6.00–13.50)abc		40.68 (25.41–102.36)a		4.11 (2.33–8.04)a	
	Luofu	14/15	7.00 (1.75–11.00)bc		74.76 (3.43–94.82)a		5.72 (1.83–10.50)a	
	Olinda	24/24	15.00 (6.00–20.00)ab		54.58 (24.20–131.18)a		4.84 (1.77–8.40)a	
	Yanxiwanlu	25/25	8.00 (4.00–13.00)abc		49.69 (23.64–82.14)a		5.06 (3.76–8.50)a	
	Rohde Red	25/25	15.00 (11.00–21.00)a		53.63 (29.18–86.02)a		3.32 (2.40–5.08)a	
	Eureka	15/15	12.00 (4.00–21.00)abc		78.92 (8.59–118.55)a		4.19 (2.66–5.67)a	
C	Yuzu	25/25	6.00 (2.50–10.50)c	0.00	66.69 (42.91–109.78)b	0.00	14.05 (8.75–19.15) ab	0.00
	Rough Lemon	21/21	10.00 (6.00–13.50)abc		71.65 (58.54–154.86)b		9. 85(7.09–11.61)b	
	Luofu	15/15	7.50 (3.75–12.00)bc		109.8 8(47.97–207.63)ab		18.33 (11.30–24.28)a	
	Olinda	24/24	18.00 (7.00–22.00)ab		131.33 (64.03–162.00)ab		9.54 (7.08–11.90)b	
	Yanxiwanlu	25/25	9.00 (4.50–14.00)abc		114.03 (87.71–189.04)ab		14.25 (9.00–19.38)ab	
	Rohde Red	25/25	17.00 (11.50–23.50)a		158.43 (99.55–212.91)a		9.32 (6.65–12.66)b	
	Eureka	15/15	13.00 (7.00–21.00)abc		206.55 (172.41–270.09)a		14.14 (10.82–19.59)ab	
pd	Yuzu	25/25	62.00 (43.5–113.5)c	0.00	482.39 (277.79–698.16)bc	0.00	6.39 (6.00–7.30)ab	0.00
	Rough Lemon	21/21	67.00 (51.50–92.50)c		357.91(287.64–514.06)c		5.80 (5.52–6.02)c	
	Luofu	15/15	66.50 (42.25–154.00)bc		472.08 (299.03–1014.46)a		6.52 (6.25–7.16)a	
	Olinda	24/24	126.00 (69.00–155.00)abc		676.63 (431.70–806.95)a		5.83 (5.34–6.48)b	
	Yanxiwanlu	25/25	98.00 (79.50–146.00)abc		684.48 (490.46–913.77)a		6.14 (5.87–6.65)ab	
	Rohde Red	25/25	130.00 (92.50–186.00)ab		827.57 (567.68–1086.85)ab		6.18 (5.84–6.50)ab	
	Eureka	15/15	175.00 (145.00–248.00)a		1109.70 (747.83–1222.02)a		5.67 (5.12–6.42)c	
E1	Yuzu	24/25	1.00 (1.00–2.00)a	0.51	1.62 (0.96–2.53)ab	0.01	1.06 (0.90–1.75)ab	0.00
	Rough Lemon	18/21	1.00 (1.00–1.00)a		0.97 (0.84–1.46)b		0.90 (0.79–1.15)c	
	Luofu	15/15	1.00 (1.00–1.25)a		2.55 (1.58–5.19)ab		2.00 (1.45–4.38)a	
	Olinda	22/24	1.00 (1.00–2.00)a		1.84 (1.03–3.12)ab		1.16(1.00–2.66)ab	
	Yanxiwanlu	21/25	1.00 (1.00–3.00)a		2.67 (0.79–6.30)ab		1.33 (0.79–3.03)ab	
	Rohde Red	21/25	1.00 (1.00–3.00)a		1.83 (0.87–5.32)ab		1.15 (0.74–2.27)ab	
	Eureka	12/15	2.00 (1.00–3.00)a		10.49 (1.76–50.69)a		5.24 (1.76–10.53)a	
E2	Yuzu	24/25	1.00 (1.00–2.00)a	0.14	264.60 (183.13–295.85)a	0.00	241.95 (80.52–295.86)a	0.00
	Rough Lemon	18/21	1.00 (1.00–1.00)a		220.73 (90.09–278.72)a		211.38 (81.39–273.13)a	
	Luofu	14/15	1.00 (1.00–1.00)a		151.31 (62.36–304.42)a		128.64 (42.39–283.11)a	
	Olinda	22/24	1.00 (1.00–2.00)a		169.26 (67.28–244.60)a		119.29 (30.09–235.39)a	
	Yanxiwanlu	21/25	1.00 (1.00–2.00)a		150.50 (39.96–237.83)ab		92.64 (18.10–195.31)ab	
	Rohde Red	21/25	1.00 (1.00–2.00)a		148.48 (68.64–215.021)ab		89.21 (26.63–180.96)ab	
	Eureka	7/15	0.00 (0.00–2.00)a		0.00 (0.00–29.48)b		0.00 (0.00–13.17)b	

PPW, proportion of individuals that produced a given waveform type; NWI, number of waveform events per insect; WDI, waveform duration (min) per insect; WDEI, waveform duration (min) per event per insect. Nonprobing (np), pathway waveforms (C), phloem salivation (E1), phloem ingestion (E2). Values presented as medians (interquartile range) along with H-statistics and Chi-square probabilities. Different letters in rows indicate significant differences between treatments at *p* < 0.05 (Kruskal–Wallis test and post hoc multiple comparisons of mean ranks for all groups—Dunn’s test). Potential drop (pd) duration is expressed in second (s).

## Data Availability

The data presented in this study are available on request from the corresponding author.

## References

[1] Zhao Z.M. (2000). Current status of research on citrus pests in China. Chin. J. Appl. Entomol..

[2] Talon M., Gmitter F.G. (2008). Citrus genomics. Int J. Plant. Genom..

[3] Brlansky R.H., Damsteegt V.D., Howd D.S., Roy A. (2003). Molecular analyses of *Citrus tristeza virus* subisolates separated by aphid transmission. Plant. Dis..

[4] Hall D.G., Albrigo L.G. (2007). Estimating the relative abundance of flush shoots in citrus with implications on monitoring insects associated with flush. HortScience.

[5] Yokomi R.K., Lastra R., Stoetzel M.B., Damsteegt V.D., Lee R.F., Garnsey S.M., Gottwald T.R., Rocha-Pena M.A., Niblett C.L. (1994). Establishment of the brown citrus aphid (Homoptera: Aphididae) in central America and the Caribbean Basin and transmission of *Citrus tristeza virus*. J. Econ. Entomol..

[6] Brlansky R.H., Roy A., Damsteegt V.D. (2011). Stem-pitting *Citrus tristeza virus* predominantly transmitted by the brown citrus aphid from mixed infections containing non-stem-pitting and stem-pitting isolates. Plant. Dis..

[7] Moreno P., Ambros S., Albiach-Marti M.R., Guerri J., Pena L. (2008). *Citrus tristeza virus*: A pathogen that changed the course of the citrus industry. Mol. Plant. Pathol..

[8] Tatineni S., Dawson W.O. (2012). Enhancement or attenuation of disease by deletion of genes from *Citrus tristeza virus*. J. Virol..

[9] Dawson W.O., Garnsey S.M., Tatineni S., Folimonova S.Y., Harper S.J., Gowda S. (2013). *Citrus tristeza virus*-host interactions. Front. Microbiol..

[10] He Y.Q., Chen W.L., Lu Z.Y., Zhou C.Y., Li Z.A., Wang X.F., Li T.S. (2014). EPG analysis of the *Toxopter acitricida* on healthy and CTV -infected citrus. J. Mt. Agric. Biol..

[11] Tang Y.Q., Weathersbee A.A., Mayer R.T. (2002). Effect of neem seed extract on the brown citrus aphid (Homoptera: Aphididae) and its parasitoid *Lysiphlebus testaceipes* (Hymenoptera: Aphidiidae). Environ. Entomol..

[12] Smaili M.C., El Ghadraoui L., Gaboun F., Benkirane R., Blenzar A. (2014). Impact of some alternative methods to chemical control in controlling aphids (Hemiptera: Sternorrhyncha) and their side effects on natural enemies on young Moroccan citrus groves. Phytoparasitica.

[13] Jeger M., Bragard C., Caffier D., Candresse T., Chatzivassiliou E., Dehnen-Schmutz K., Gilioli G., Gregoire J.C., Jaques Miret J.A., EFSA Panel on Plant Health (PLH) (2018). Pest categorization of *Toxoptera citricida*. EFSA J..

[14] Zhou H., Du L., Wan F.H., Zhou H. (2020). Comparative analysis of stylet penetration behaviors of *Eriosoma lanigerum* (Hemiptera: Aphididae) on main apple cultivars in China. J. Econ. Entomol..

[15] Pobozniak M., Gaborska M., Wojtowicz T. (2021). Resistance and tolerance of ten carrot cultivars to the hawthorn-carrot aphid, *Dysaphis crataegi* Kalt., in Poland. PLoS ONE.

[16] Bhumannavar B.S., Singh S.P., Sulladmath V.V. (1989). Field evaluation of citrus germplasm for resistance to the black aphid, *Toxoptera aurantii* (Boy.) under tropical humid South Indian conditions. Insect Sci. Applic..

[17] Tsai J.H. (1998). Development, survivorship, and reproduction of *Toxoptera citricida* (Kirkaldy) (Homoptera: Aphididae) on eight host plants. Environ. Entomol..

[18] Lu Z.Y., Chen W.L., He Y.Q., Zhou C.Y., Wang X.F., Li T.S. (2014). Difference in feeding behavior of brown citrus aphid *Toxoptera citricida* on five species of orange seedlings. J. Plant. Protect..

[19] Kimmins F.M., Tjallingii W.F. (1985). Ultrastructure of sieve element penetration by aphid stylets during electrical recording. Exp. Appl..

[20] Tjallingii W.F., Hogen Esch T. (1993). Fine structure of aphid stylet routes in plant tissues in correlation with EPG signals. Physiol. Entomol..

[21] Prado E., Tjallingii W.F. (1994). Aphids activities during sieve element punctures. Exp. Appl..

[22] Chen J.Q., Martín B., Rahbé Y., Fereres A. (1997). Early intracellular punctures by two aphid species on near-isogenic melon lines with and without the virus aphid transmission (Vat) resistance gene. Eur. J. Plant Pathol..

[23] Tjallingii W.F., Gabrys B. (1999). Anomalous stylet punctures of phloem sieve elements by aphids. Exp. Appl..

[24] Jimenez J., Garzo E., Alba-Tercedor J., Moreno A., Fereres A., Walker G.P. (2019). The phloem pd: A distinctive brief sieve element stylet puncture prior to sieve element phase of aphid feeding behavior. Arthropod Plant Interact..

[25] Van Helden M., Tjallingii W.F. (1993). Tissue localization of lettuce resistance to the aphid *Nasonovia ribisnigri* using electrical penetration graphs. Exp. Appl..

[26] Zhao M., Tian T.W., Li W.Z., Luo M.H., Guo X.R., Yan F.M. (2015). Comparative analysis of *Rhopalosiphum maidis* feeding behaviors on eight maize hybrids (inbreds). Sci. Agr. Sinica.

[27] Peng H.C., Walker G.P. (2018). Sieve element occlusion provides resistance against *Aphis gossypii* in TGR-1551 melons. Insect Sci..

[28] Garzo E., Soria M.L., Gómez-Guillamon M.L., Fereres A. (2002). Feeding behaviour of *Aphis gossypii* on resistant accessions of different melon genotypes (*Cucumis melo*). Phytoparasitica.

[29] Liang L.Y., Liu L.F., Yu X.P., Han B.Y. (2012). Evaluation of the resistance of different tea cultivars to tea aphids by EPG technique. J. Integr. Agr..

[30] Alvarez A.E., Broglia V.G., Alberti D’Amato A.M., Wouters D., van der Vossen E., Garzo E., Tjallingii W.F., Dicke M., Vosman B. (2012). Comparative analysis of *Solanum stoloniferum* responses to probing by the green peach aphid *Myzus persicae* and the potato aphid *Macrosiphum euphorbiae*. Insect Sci..

[31] Sun M., Voorrps R.E., Steenhuis-Broers G., van’t Westende W., Vosman B. (2018). Reduced phloem uptake of *Myzus persicae* on an aphid resistant pepper accession. BMC Plant Biol..

[32] Guo J.G., Yuan W.N., Zhou T.W., Zhang X.R., He C.G., Zhao G.Q., Zhang Z.W. (2017). Feeding behavior of biotype E of the greenbug, *Schizaphis graminum* (Hemiptera:Aphididae) on oats with different phenotypic resistance. Acta Entomol. Sinica.

[33] Jiang Y.X., Chang W.J., Zhan Y.D., Liu Z., Liu Y. (2020). Investigation on the resistance of wheat germplasm resources to aphid based on fuzzy recognition and electrical penetration graph (EPG) techniques. Chin. J. Appl. Ecol..

[34] Tjallingii W.F. (1978). Electronic recording of penetration behaviour by aphids. Entomol. Exp. Appl..

[35] Spiller N.J., Koenders L., Tjallingii W.F. (2011). Xylem ingestion by aphids—A strategy for maintaining water balance. Entomol. Exp. Appl..

[36] Garzo E., Moreno A., Plaza M., Fereres A. (2021). Feeding behavior and virus-transmission ability of insect vectors exposed to systemic insecticides. Plants.

[37] Sarria E., Cid M., Garzo E., Fereres A. (2009). Excel workbook for automatic parameter calculation of EPG data. Comput. Electron. Agr..

[38] Schwarzkopf A., Rosenberger D., Niebergall M., Gershenzon J., Kunert G. (2013). To feed or not to feed: Plant factors located in the epidermis, mesophyll, and sieve elements influence pea aphid’s ability to feed on Legume species. PLoS ONE.

[39] Zhao R.N., He Y.Q., Lu Z.Y., Chen W.L., Zhou C.Y., Wang X.F., Li T.S. (2019). An analysis of the feeding behavior of three stages of *Toxoptera citricida* by DC electrical penetration graph waveforms. Entomol. Exp. Appl..

[40] Powell C.A., Burton M.S., Pelosi R.R., Rundell P.A., Ritenour M.A., Bullock R.C. (2006). Six-year evaluation of brown citrus and spirea aphid populations in a citrus grove and the effects of insecticides on these populations. HortScience.

[41] Zehnder C.B., Hunter M.D. (2008). Effects of nitrogen deposition on the interaction between an aphid and its host plant. Ecol. Entomol..

[42] Ford E.S. (1942). Anatomy and histology of the eureka lemon. Bot. Gaz..

[43] Wagner G.J., Wang E., Shepherd R.W. (2004). New approaches for studying and exploiting an old protuberance, the plant trichome. Ann. Bot..

[44] Simmons A.T., Gurr G.M., Mcgrath D., Nicol H.I., Martin P.M. (2003). Trichomes of *Lycopersicon* spp. and their effect on *Myzus persicae* (Sulzer) (Hemiptera: Aphididae). Aust. J. Entomol..

[45] Rakha M., Hanson P., Ramasamy S. (2017). Identification of resistance to *Bemisia tabaci* Genn. in closely related wild relatives of cultivated tomato based on trichome type analysis and choice and no-choice assays. Genet. Resour. Crop. Evol..

[46] Rodriguez-Lopez M.J., Garzo E., Bonani J.P., Fernandez-Munoz R., Moriones E., Fereres A. (2012). Acylsucrose-producing tomato plants forces *Bemisia tabaci* to shift its preferred settling and feeding site. PLoS ONE.

[47] Dardouri T., Gomez L., Ameline A., Costagliola G., Schoeny A., Gautier H. (2021). Non-host volatiles disturb the feeding behavior and reduce the fecundity of the green peach aphid, *Myzus persicae*. Pest. Manag. Sci..

[48] Walker G.P. (1988). The role of leaf cuticle in leaf age preference by bayberry whitefly (Homoptera: Aleyrodidae) on lemon. Ann. Entomol. Soc. Am..

[49] Prado E., Tjallingii W.F. (1997). Effects of previous plant infestation on sieve element acceptance by two aphids. Entomol. Exp. Appl..

[50] Nalam V.J., Han J.L., Pitt W.J., Acharya S.R., Nachappa P. (2021). Location, location, location: Feeding site affects aphid performance by altering access and quality of nutrients. PLoS ONE.

[51] Shugart H., Ebert T., Gmitter F., Rogers F. (2019). The power of electropenetrography in enhancing our understanding of host plant-vector interactions. Insects.

[52] Wensler R.J., Filshie B.K. (1969). Gustatory sense organs in the food canal of aphids. J. Morphol..

[53] Kordan B., Stec K., Slominski P., Giertych M.J., Wróblewska-Kurdyk A., Gabrys B. (2018). Susceptibility of forage legumes to infestation by the pea aphid *Acyrthosiphon pisum* (Harris) (Hemiptera: Aphididae). Crop. Pasture Sci..

[54] Medina-Ortega K.J., Walker G.P. (2013). Does aphid salivation affect phloem sieve element occlusion in vivo?. J. Exp. Bot..

[55] Caillaud C.M., Niemeyer H.M. (1996). Possible involvement of the phloem sealing system in the acceptance of a plant as host by an aphid. Experientia.

[56] Miao J., Han B.Y., Zhang Q.H. (2014). Probing behavior of *Empoasca vitis* (Homoptera: Cicadellidae) on resistance and susceptible cultivars of tea plants. J. Insect Sci..

[57] Ng J.C.K., Perry K.L. (2004). Transmission of plant viruses by aphid vectors. Mol. Plant. Pathol..

[58] Rodríguez-López M.J., Garzo E., Fereres A., Fernández-Muñoz R., Bonani J.P., Moriones E. (2011). Whitefly resistance traits derived from the wild tomato *Solanum pimpinellifolium* affect the preference and feeding behavior of *Bemisia tabaci* and reduce the spread of Tomato yellow leaf curl virus. Phytopathology.

[59] Paprocka M., Gliszczyńska A., Dancewicz K., Gabryś B. (2018). Novel hydroxy- and epoxy-cis-jasmone and dihydro jasmone derivatives affect the foraging activity of the peach potato aphid *Myzus persicae* (Sulzer) (Homoptera: Aphididae). Molecules.

[60] Escudero-Martinez C., Leybourne D.J., Bos J.I.B. (2021). Plant resistance in different cell layers affects aphid probing and feeding behaviour during non-host and poor-host interactions. B Entomol. Res..

[61] Lucini T., Panizzi A.R., Bueno A.D.F. (2021). Evaluating resistance of the soybean block technology cultivars to the Neotropical brown stink bug, *Euschistus heros* (F.). J. Insect Physiol..

[62] Hao Z.P., Zhan H.X., Wang Y.L., Hou S.M. (2019). How cabbage aphids *Brevicoryne brassicae* (L.) make a choice to feed on *Brassica napus* cultivars. Insects.

